# Transcutaneous Spinal Cord Direct-Current Stimulation Modulates Functional Activity and Integration in Idiopathic Restless Legs Syndrome

**DOI:** 10.3389/fnins.2020.00873

**Published:** 2020-08-21

**Authors:** Min Zeng, Li Wang, Bochao Cheng, Guiqiang Qi, Junwei He, Zhexue Xu, Tao Han, Chunyan Liu, Yuping Wang

**Affiliations:** ^1^Department of Radiology, Pidu District People’s Hospital, Chengdu, China; ^2^Department of Neurology, Xuanwu Hospital, Capital Medical University, Beijing, China; ^3^Beijing Key Laboratory of Neuromodulation, Beijing, China; ^4^Department of Radiology, West China Second University Hospital of Sichuan University, Chengdu, China

**Keywords:** idiopathic RLS, tsDCS, resting state, fMRI, therapy

## Abstract

Idiopathic restless legs syndrome (RLS) is a sensorimotor disorder and is suggested to be caused by central nervous system abnormalities. Non-invasive transcutaneous spinal direct-current stimulation (tsDCS) was recently used for RLS therapy. However, the neurophysiological basis of tsDCS treatment is still unknown. In this study, we explored the neural basis of tsDCS in 15 RLS patients and 20 gender- and age-matched healthy controls using resting-state functional magnetic resonance imaging. We calculated the whole-brain voxel-wise fractional amplitude of low-frequency fluctuations (fALFF), regional homogeneity (ReHo), and weighted degree centrality (DC) to characterize the intrinsic functional activities and the local and global functional integration. We found that tsDCS can effectively improve the sleep and RLS symptoms in RLS patients. Moreover, after tsDCS therapy, the RLS patients showed decreased fALFF in the right anterior insula/temporal pole, decreased ReHo in the supplementary motor area, increased weighted DC in the left primary visual cortex, and decreased weighted DC in the right posterior cerebellum. The changed patterns were consistent with that found between RLS patients and healthy controls. The weighted DC in the left primary visual cortex after treatment and the fALFF in the right anterior insula/temporal pole before treatment were significantly and marginally correlated with sleep and RLS symptom scores, respectively. These results revealed that tsDCS can normalize the functional patterns of RLS patients and is an effective way for RLS therapy. Our findings provide the neurophysiological basis for tsDCS treatment and may facilitate understanding the neuropathology of RLS and directing other neuromodulation treatments.

## Introduction

The restless legs syndrome (RLS) is a common sensorimotor disorder, and the prevalence of the syndrome is between 3 and 9% in the general population (Phillips et al., 2000; Rothdach et al., 2000). The main characteristic of RLS is an unpleasant sensation in the lower limbs, especially during nighttime. RLS is primarily associated with the underlying genetics, iron deficiency, uremia, pregnancy, or end-stage renal disease (Trenkwalder et al., 2005). Dopaminergic medication is the main therapeutic approach for RLS, but given augmentation and the side effects of medication (Paulus and Trenkwalder, 2006; Earley and Silber, 2010; Allen et al., 2011; García-Borreguero and Williams, 2011), currently, non-pharmacological neuromodulation therapy has become a more and more popular approach for brain disorder treatment as it has less side effects.

A few previous studies have revealed that central nervous system overactivity may be the underlying mechanism of RLS (Bucher et al., 1997; Trenkwalder et al., 2005; Spiegelhalder et al., 2008; Margariti et al., 2012; Liu et al., 2015, 2018). A recent study using the non-invasive and painless transcutaneous spinal direct-current stimulation (tsDCS) technique demonstrated that tsDCS can reduce spinal cord excitability and can effectively alleviate the symptoms of RLS patients (Heide et al., 2014). This finding indicated that tsDCS may open a new avenue for RLS therapy. However, the neurophysiological basis of tsDCS therapy for RLS remains unclear.

Resting-state functional magnetic resonance imaging (rs-fMRI) mainly characterizes the spontaneous low-frequency fluctuations of brain activities as measured by blood oxygen level-dependent signals (Fox and Raichle, 2007). rs-fMRI has been widely used to detect the intrinsic functional organization and functional couplings in healthy subjects and in diseases (Fox et al., 2006; Wang et al., 2015, 2016b,2019b; Wang C. et al., 2017; Wang J. et al., 2017; Sun et al., 2018; Xu et al., 2019; Gao et al., 2020). Therefore, rs-fMRI is a particularly effective way to explore the neurophysiological basis of neuromodulation treatment for brain disorders (Liu et al., 2015; Wang et al., 2016a, 2018, 2020). To characterize the functional activity and the interaction patterns during resting, the amplitude of low-frequency fluctuations (ALFF), functional signals regional homogeneity (ReHo), and weighted degree centrality (DC) were proposed to delineate the functional oscillation (Zang et al., 2007), local information integration (Zang et al., 2004), and global functional integration (Wu et al., 2016; Liu et al., 2018; Song et al., 2019), respectively. Therefore, ALFF, ReHo as well as DC can provide a comprehensive and complementary evidence for the neurophysiological basis of tsDCS treatment.

In this study, we aimed to identify the neurophysiological basis of tsDCS treatment for RLS using rs-fMRI in 15 RLS patients before and after tsDCS and in 20 age- and gender-matched healthy controls. First, voxel-wise fALFF, ReHo, and weighted DC maps for each subject were calculated. Then, paired *t*-tests were used to identify the changed functional activity and local and global information integration in RLS patients after tsDCS. Finally, correlation analyses were performed to reveal the relationships between changed neural measurements and clinical performances.

## Materials and Methods

### Participants

Thirty idiopathic RLS patients (23 females and seven males; mean age: 62.1 ± 8.04 years) and 20 gender- and age-matched healthy controls (17 females and 3 males; mean age: 60.1 ± 7.9 years) were used in our current study. The RLS patients were diagnosed based on the International Criteria of the International Restless Legs Syndrome Study Group. Patients with anemia, renal disease, a history of alcohol or drug abuse, spinal cord injury, pregnancy, or other neuropathies were excluded. All patients were randomly divided into the tsDCS stimulation group and the sham stimulation group, with 15 patients in each group. The severity of RLS was characterized using the International RLS Rating Scale (IRLS-RS) and Pittsburgh Sleep Quality Index (PSQI). Both IRLS-RS and PSQI were measured at baseline (before treatment) and at 2 weeks after treatment. The 20 right-handed healthy controls were recruited from the community and did not have any neurological disorders nor were taking medication and did not have a family history of RLS. The written informed consents were provided and obtained from all subjects. The study was approved by the ethics committee of the Xuan Wu Hospital of Capital Medical University and was in accordance with the Helsinki Declaration and its later amendments or comparable ethical standards.

### The tsDCS Procedures

The tsDCS protocol was performed according to that reported in a previous study (Heide et al., 2014). Anodal stimulation with a constant direct current was conducted. The subjects were placed in prone position. The center of the stimulation electrode was placed over the 10th thoracic vertebra and the return electrode was placed above the right shoulder. Both equally sized rectangular electrodes (5 × 7 × 6 cm) were applied. The stimulation current strength was 2 mA for 20 min. A current density of 0.057 mA/cm^2^ and a total delivered charge of 51.3 mC/cm^2^, which is far below the threshold for tissue damage, were applied (Nitsche et al., 2003; Liebetanz et al., 2009). The treatment was applied daily for 14 consecutive days for all the patients. Sham stimulation was delivered by turning off the stimulator after 30 s, and the subjects experienced the same tingling sensation at the beginning of the stimulus.

### Resting-State fMRI Data Collection

The resting-state fMRI data were collected using a Siemens Trio 3-Tesla (Siemens, Erlangen, Germany) head MRI scanner. All the RLS patients were scanned twice, i.e., before and after treatment, and the healthy controls were only scanned once. All the subjects were asked not to move as much as possible and to relax their minds and to keep their eyes closed without falling asleep. Foam pads and headphones were used to reduce head movement and scanner noise. The resting-state fMRI data were collected with the following parameters: repetition time/echo time ratio = 2,000/30 ms, flip angle = 90°, acquisition matrix size = 64 × 64, voxel size = 3.75 mm × 3.75 mm × 4 mm with 1-mm gap, 33 slices, and 240 volumes.

### Resting-State fMRI Data Preprocessing

The resting-state fMRI data for each subject were preprocessed using SPM8 software with DPARSF, version 2.3^1^. The first 10 volumes were discarded to facilitate magnetization equilibrium effects; the remaining images were realigned to the first volume to correct head motion; the fMRI images were then normalized to EPI template in standard MNI space and resampled to 3-mm isotropic voxels; Friston 24-parameter model of head motion, white matter, cerebrospinal fluid, and global mean signals were regressed out and filtered with a temporal band-path of 0.01–0.1 Hz. To further exclude the head motion effects, first, if the head motion exceeds 3 mm or 3°, the subject was excluded. Under this criterion, no subjects were excluded. Second, a scrubbing method was conducted to censor each subject’s bad images with the mean frame displacement (FD) above 0.5 mm, and one volume before and two volumes after the bad volume were discarded (Power et al., 2012). The voxel-wise fALFF was calculated for each subject before the preprocessing of filtering, and the ReHo and weighted degree centrality were calculated with the scrubbed fMRI data.

### The fALFF, ReHo, and Weighted DC Calculation

The fALFF was used to characterize the resting-state functional activity of each brain voxel since fALFF is more robust to white matter and cerebrospinal fluid noises and can better exclude individual variations compared with ALFF (Zou et al., 2008). To calculate fALFF, the time series for each voxel was transformed to the frequency domain and the power spectrum was obtained. fALFF is defined as the power within the low-frequency range (0.01–0.1 Hz) divided by the total power in the entire detectable frequency range. To characterize the local functional integration, voxel-wise ReHo was mapped for each participant by calculating the Kendall’s coefficient of concordance for the time series of a given voxel with those of its nearest 26 neighbors (Zang et al., 2004). The global functional integration defined by weighted DC was also calculated for each subject at voxel-wise level. To calculate the weighted DC, functional connections of a given voxel and all other voxels constrained within a binary gray matter mask, created by thresholding the gray matter probability template with 0.2 in the brain, were first calculated (Wang et al., 2014; Wu et al., 2016; Liu et al., 2018). Then, the weighted DC for each voxel was obtained by averaging the functional connectivity strengths higher than a threshold of 0.25 over the whole brain (Wang et al., 2014; Wu et al., 2016; Liu et al., 2018). Finally, the fALFF, ReHo, and weighted DC maps were transformed to *Z* scores to improve normality. For statistical analyses, all these measurements were spatially smoothed with a 6-mm Gaussian kernel. Paired *t*-tests were performed to compare the fALFF, ReHo, and weighted DC maps between RLS patients before and after tsDCS. The significance was determined using a Gaussian random field correction method with *p* < 0.05. Moreover, the regional mean fALFF, ReHo, and weighted DC in RLS patients before and after tsDCS and in healthy controls were calculated. Two-sample *t*-tests were conducted between healthy controls and RLS patients before and after tsDCS was performed. The significant level was set at *p* < 0.05 with Bonferroni correction.

### Correlation Analyses

To explore the relationship between the resting-state fMRI indices and the clinical characteristics, correlation analyses were performed between the mean fALFF, ReHo, weighted DC, and PSQI and IRLS-RS scores in RLS patients before, after, and after-minus-before. The significance was set at *p* < 0.05.

## Results

### Clinical Characteristics

A chi-square test and two-sample *t*-test were performed for gender and age, respectively. There were no significant differences in gender (*p* = 0.15), and age (*p* = 0.64) between RLS patients and healthy controls (Table 1). Paired *t*-tests were performed for clinical characteristics, and it was found that the RLS patients showed significantly decreased IRLS-RS (*p* < 10^–6^) and PSQI (*p* < 10^–4^) scores after tsDCS compared with before treatment (Table 1). In the sham tsDCS group, no significant changes in IRLS-RS (*p* = 0.22) and PSQI (*p* = 0.50) scores were found after treatment (Table 1).

**TABLE 1:** Demographics and clinical characteristics of the subjects used in present study.

	RLS			
	
	tsDCS	Sham	HC	
Subjects	(*n* = 15)	(*n* = 15)	(*n* = 20)	*p-*value
Gender (male: female)	3/12	4/11	3/17	>0.05
Age (mean ± SD)	61.4 ± 8.4	62.9 ± 7.8	60.1 ± 7.9	>0.05
IRLS-RS scores (mean ± SD)				5.09 × 10^–6,a^, 0.22^*b*^
Before	27.2 ± 6.7	24.6 ± 7.8		
After	15.0 ± 7.4	23.4 ± 7.2		
PSQI scores (mean ± SD)				1.09 × 10^–5,a^, 0.50^*b*^
Before	14.9 ± 2.8	12.3 ± 3.4		
After	10.3 ± 3.7	11.9 ± 3.6		

*A Pearson chi-squared test was used for gender comparison. Independent two-sample *t*-tests were used for age comparisons. The paired *t*-tests were used for clinical assessment comparisons before and after treatment. RLS-tsDCS, Restless legs syndrome patients receiving transcutaneous spinal cord direct current stimulation; RLS-sham, Restless legs syndrome patients sham stimulation; HC, healthy control; IRLS-RS, International RLS Rating Scale; PSQI, Pittsburgh Sleep Quality Index. The “a” represents the comparison between RLS patients before and after tsDCS treatment; the “b” represents the comparison between RLS patients before and after sham treatment.*

### Changed fALFF After tsDCS

**FIGURE 1:**
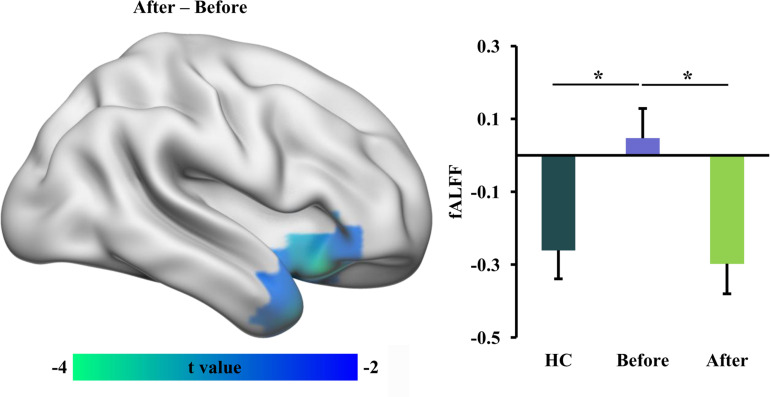
Decreased fractional amplitude of low-frequency fluctuation (fALFF) in restless leg symptom (RLS) patients after transcutaneous spinal cord direct current stimulation (tsDCS). Paired *t*-test was performed and identified significantly decreased fALFF in right anterior insula (INS)/temporal pole (TP) in RLS patients after tsDCS compared to that before tsDCS. Regional mean fALFF values in the right anterior INS/TP were calculated in healthy controls (HC) and RLS patients before and after tsDCS, and two-sample *t*-tests were performed between HC and RLS patients before and after tsDCS and found decreased fALFF in HC compared to RLS patients before tsDCS treatment. No significant difference in fALFF between HC and RLS patients after tsDCS was found. *p < 0.05.

Statistical analyses identified a significant difference in functional activity, characterized using fALFF in RLS patients before and after tsDCS. Significantly reduced fALFF in the right anterior insula/temporal pole (INS/TP) was identified in RLS patients after tsDCS compared to before tsDCS treatment (Table 2 and Figure 1). Compared with healthy controls, the RLS patients also showed elevated fALFF in the right INS/TP (Figure 1). There was no significant difference in fALFF between healthy controls and RLS patients after tsDCS treatment (Figure 1).

**TABLE 2:** Regions with changed fALFF, ReHo, and weighted DC in RLS patients after tsDCS treatment.

			Peak MNI			
			
			coordinates			
Indices	Brain regions	L/R	X	Y	Z	*t* values
fALFF	Anterior insula/temporal pole	R	36	24	–18	–5.5
ReHo	Supplementary motor area	L	–3	–15	57	–4.3
Weighted DC	Primary visual cortex	L	–12	–87	15	4.2
	Posterior cerebellum	L	–24	–30	–57	–4.4

*RLS, restless leg symptom; tsDCS, transcutaneous spinal cord direct current stimulation; MNI, Montreal Neurological Institute; fALFF, fractional amplitude of low frequency fluctuations; ReHo, regional homogeneity; DC, degree centrality; L, left hemisphere; R, right hemisphere.*

### Changed ReHo After tsDCS

**FIGURE 2:**
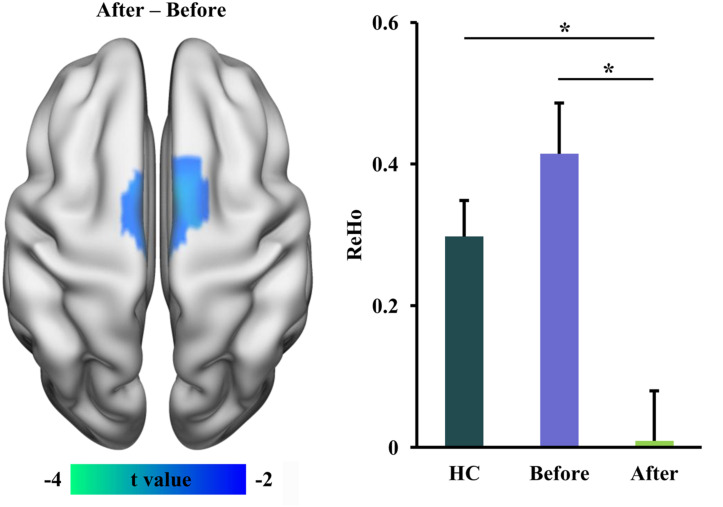
Decreased regional homogeneity (ReHo) in restless leg symptom (RLS) patients after transcutaneous spinal cord direct current stimulation (tsDCS) treatment. Paired *t*-test found significantly decreased ReHo in supplementary motor area (SMA) in RLS patients after tsDCS compared to that before tsDCS. Regional mean ReHo values in SMA were calculated in healthy controls (HC) and RLS patients before and after tsDCS. Two-sample *t*-tests also found significantly decreased ReHo in RLS patients after tsDCS treatment compared to HC. No significant difference in ReHo between HC and RLS patients before tsDCS was found. *p < 0.05.

We used ReHo measurement to characterize local functional integration and found decreased ReHo in supplementary motor area (SMA) in RLS patients after tsDCS treatment (Table 2 and Figure 2). A similar pattern was also found between healthy controls and RLS patients before tsDCS treatment, but no significant difference was found (Figure 2). Furthermore, we found a significant difference in ReHo between healthy controls and RLS patients after tsDCS (Figure 2).

### Changed Weighted DC After tsDCS

**FIGURE 3:**
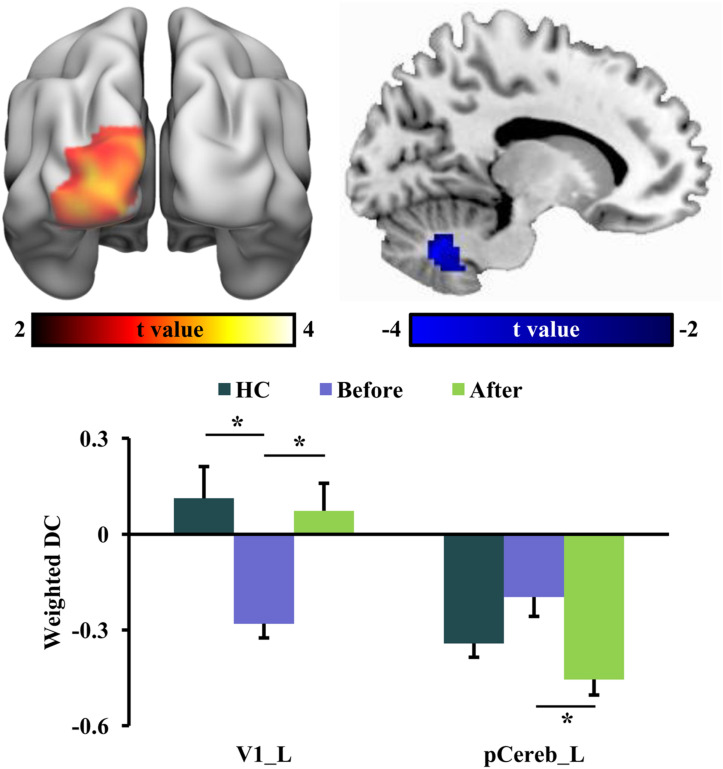
Changed weighted degree centrality (DC) in restless leg symptom (RLS) patients after transcutaneous spinal cord direct current stimulation (tsDCS) treatment. Paired *t*-test found significantly decreased weighted DC in posterior cerebellum (pCereb) and significantly increased weighted DC in primary visual cortex (V1) in RLS patients after tsDCS compared to that before tsDCS. Regional mean weighted DC values in pCereb and V1 were calculated in healthy controls (HC) and RLS patients before and after tsDCS. Two-sample *t*-tests only found significantly increased weighted DC of V1 in HC compared to RLS patients before tsDCS. No other significant differences in weighted DC between HC and RLS patients before or after tsDCS were found. *p < 0.05.

The global integration was characterized using weighted DC in this study. In RLS patients after tsDCS, increased weighted DC was shown in the left primary visual cortex (V1) and decreased weighted DC was shown in the posterior cerebellum (pCereb) (Table 2 and Figure 3). The increased weighted DC in the left V1 was also found in healthy controls compared to RLS patients before tsDCS (Figure 3). In RLS patients after tsDCS, the increased and the decreased weighted DC patterns were similar with that in healthy controls, but no differences were found between healthy controls and RLS patients after tsDCS (Figure 3).

### Correlation Analyses

**FIGURE 4:**
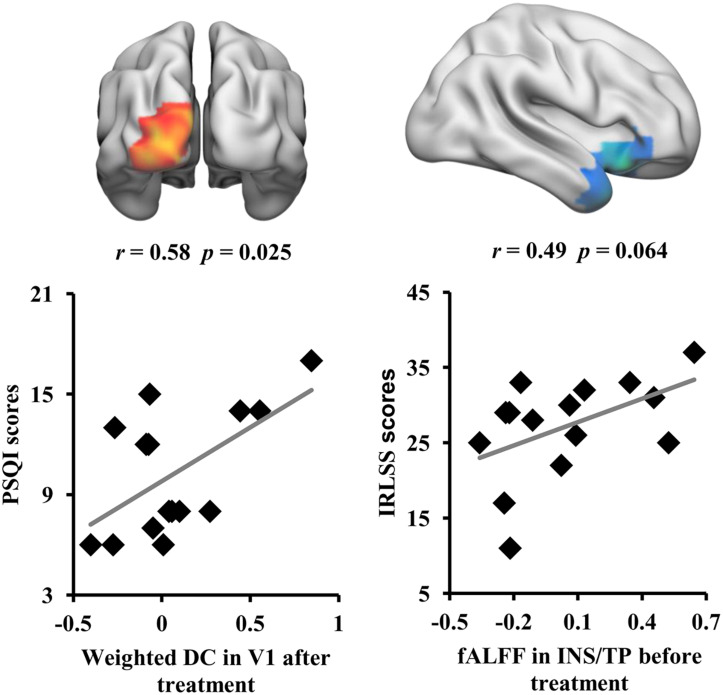
Correlation analyses between neuroimaging indices and clinical characteristics. A significant correlation was found between the weighted DC values of V1 in restless leg symptom (RLS) patients after transcutaneous spinal cord direct current stimulation (tsDCS) treatment and Pittsburgh Sleep Quality Index scores. A marginal correlation between the fractional amplitude of low-frequency fluctuation values of the right anterior insula/temporal pole in RLS patients before tsDCS treatment and the international RLS Rating Scale scores was found.

Correlation analyses revealed that the weighted DC values of the left V1 in RLS patients after tsDCS treatment were significantly correlated with the PSQI scores (*r* = 0.58, *p* = 0.025) (Figure 4). Moreover, a marginal correlation between the fALFF values in the right anterior INS/TP and the IRLS-RS scores in RLS patients before tsDCS treatment was found (*r* = 0.49, *p* = 0.064) (Figure 4).

## Discussion

In this study, we revealed that tsDCS can effectively alleviate RLS symptoms by modulating the low-frequency oscillation and the local and global information integration. We found decreased fALFF in the right anterior INS/TP, decreased ReHo in SMA, and increased weighted DC in the left V1 as well as decreased weighted DC in the posterior cerebellum in RLS patients after tsDCS treatment. Moreover, the weighted DC values in the left V1 and the fALFF values in the right anterior INS/TP were significantly or marginally correlated with the PSQI and the IRLS-RS scores, respectively. Our findings demonstrate that tsDCS can normalize the neuronal activities for effective treatment of RLS, which provide the preliminary evidence for the neurophysiological basis of tsDCS treatment for RLS.

Using resting-state fMRI, we found that tsDCS treatment reduced the low-frequency functional oscillation in the right anterior INS/TP. The insula serves as an interface between internal and extra-personal stimuli and thus plays an important role in integrating emotion, cognitive, and homeostatic information (Craig, 2011; Simmons et al., 2013). The anterior INS primarily participates in cognitive control, decision making, and emotion and pain processing through connections with the dorsal anterior and posterior cingulate cortex (Dosenbach et al., 2007; Thielscher and Pessoa, 2007; Kelly et al., 2012). A large number of recent studies have revealed that the functional disruption of the anterior INS results in depression and the functional remodeling is closely related to depression remission (Wang C. et al., 2017; Wang et al., 2019a, b). Moreover, the previous functional mapping of TP also identified the emotion processing of this area (Olson et al., 2007; Fan et al., 2014). Thus, decreased fALFF in the anterior INS/TP may contribute to the cognitive and emotional recovery in RLS patients after tsDCS treatment. Given the correlation result between the fALFF of the anterior INS/TP and the IRLS-RS scores and the role of pain processing in the anterior INS (Kong et al., 2006), the decreased fALFF in the anterior INS suggested that tsDCS may alleviate the RLS symptoms by reducing the sensitivity of pain threshold.

We also found decreased ReHo in SMA and decreased weighted DC in the posterior cerebellum in RLS patients after tsDCS treatment. These findings suggested that tsDCS treatment can reduce the local and global functional integration in RLS patients. SMA and cerebellum are the main parts of the sensorimotor circuit in the brain (Goldberg, 2010; Buckner, 2013). Previous fMRI studies have identified an overactivity of the cerebellum during sensory discomfort and periodic limb movements (Bucher et al., 1997; Spiegelhalder et al., 2008; Margariti et al., 2012). Our recent study using resting-state fMRI also identified an increased functional connectivity between the cerebellum and the thalamus in RLS patients (Liu et al., 2018). The SMA plays an important role in the planning of complex movements (Goldberg, 2010). Liu et al. (2015) found that RLS patients showed a lower ALFF value in SMA, and repetitive transcranial magnetic stimulation induced an increase in the functional activity of this area. In our study, we found that effective tsDCS treatment can decrease the local and global functional integration of SMA and the cerebellum, which indicated that the functional segregation of SMA and the reduced excessive activity of the cerebellum are important for alleviating the RLS symptoms and may be the neuropathology of RLS. Notably, we found that the ReHo in SMA is lower in RLS patients after treatment compared to both in healthy controls and in RLS patients before treatment. This finding indicated that lower ReHo in SMA may be a biomarker for remission of RLS symptoms. However, no significant differences in the ReHo of SMA between RLS patients before treatment and healthy controls were observed, which may mainly result from the small sample of RLS patients and thus with low statistical power.

Finally, we found that tsDCS treatment increased the global functional integration of V1 in RLS patients. V1 is important for visual information processing. Recent functional MRI and lesion-related studies in humans have revealed that V1 is involved in visuospatial attention and conscious visual awareness (Silvanto et al., 2005; Fang et al., 2008; Wang et al., 2016a). Since motor responses are closely linked to visual stimuli, thus, visual information processing in V1 is an important part of the sensorimotor network. Moreover, a previous study reported that stimulation of the occipital cortex has an antinociceptive effect by activating the descending inhibitory pathway (Zhang et al., 2007). In our study, besides the increased global functional integration, we also found that the weighted DC in V1 is closely associated with the PSQI scores. Our findings, together with previous results, indicated that the increased global information integration of V1 after tsDCS may serve as an effective biomarker of treatment response.

Several limitations of the present study are worth mentioning. First, only 15 RLS patients were used to investigate the changes of functional activity and local and global information integration. Thus, these findings should be further validated with a larger sample. Second, our study only focused on voxel-wise functional characterization; how tsDCS modulates the topological architecture of the functional network should be explored in future studies.

## Conclusion

The present study revealed the voxel-wise functional changes in low-frequency oscillation and local and global information integration in RLS patients after tsDCS treatment. These results showed that the sensorimotor areas and the visual cortex are important biomarkers for response of RLS treatment. Our findings provide a comprehensive evidence for the neurophysiological basis of tsDCS treatment for RLS patients from different aspects and will facilitate the future understanding of the neuropathology of RLS and the direction of therapy for RLS patients.

## Data Availability Statement

The raw data supporting the conclusions of this article will be made available by the authors, without undue reservation.

## Ethics Statement

The studies involving human participants were reviewed and approved by Xuan Wu Hospital of Capital Medical University. The patients/participants provided their written informed consent to participate in this study.

## Author Contributions

All authors listed have made a substantial, direct and intellectual contribution to the work, and approved it for publication.

## Conflict of Interest

The authors declare that the research was conducted in the absence of any commercial or financial relationships that could be construed as a potential conflict of interest.

## Footnotes

1 www.restfmri.net/forum/DPARSF
